# A Quality-of-Service-Aware Service Composition Method in the Internet of Things Using a Multi-Objective Fuzzy-Based Hybrid Algorithm

**DOI:** 10.3390/s23167233

**Published:** 2023-08-17

**Authors:** Marzieh Hamzei, Saeed Khandagh, Nima Jafari Navimipour

**Affiliations:** 1 Department of Computer Engineering, Tabriz Branch, Islamic Azad University, Tabriz 51376-53515, Iran; 2 Electrical Engineering Department, Tabriz Branch, University of Applied Sciences and Technology, Tabriz 51376-53515, Iran; 3 Department of Computer Engineering, Faculty of Engineering and Natural Sciences, Kadir Has University, 34083 Istanbul, Turkey; 4 Future Technology Research Center, National Yunlin University of Science and Technology, Douliou 64002, Taiwan

**Keywords:** Internet of Things (IoT), service, composition, heuristic algorithm, cloud computing, fog computing, service composition, meta-heuristic algorithm, ABC, ACO, fuzzy logic

## Abstract

The Internet of Things (IoT) represents a cutting-edge technical domain, encompassing billions of intelligent objects capable of bridging the physical and virtual worlds across various locations. IoT services are responsible for delivering essential functionalities. In this dynamic and interconnected IoT landscape, providing high-quality services is paramount to enhancing user experiences and optimizing system efficiency. Service composition techniques come into play to address user requests in IoT applications, allowing various IoT services to collaborate seamlessly. Considering the resource limitations of IoT devices, they often leverage cloud infrastructures to overcome technological constraints, benefiting from unlimited resources and capabilities. Moreover, the emergence of fog computing has gained prominence, facilitating IoT application processing in edge networks closer to IoT sensors and effectively reducing delays inherent in cloud data centers. In this context, our study proposes a cloud-/fog-based service composition for IoT, introducing a novel fuzzy-based hybrid algorithm. This algorithm ingeniously combines Ant Colony Optimization (ACO) and Artificial Bee Colony (ABC) optimization algorithms, taking into account energy consumption and Quality of Service (QoS) factors during the service selection process. By leveraging this fuzzy-based hybrid algorithm, our approach aims to revolutionize service composition in IoT environments by empowering intelligent decision-making capabilities and ensuring optimal user satisfaction. Our experimental results demonstrate the effectiveness of the proposed strategy in successfully fulfilling service composition requests by identifying suitable services. When compared to recently introduced methods, our hybrid approach yields significant benefits. On average, it reduces energy consumption by 17.11%, enhances availability and reliability by 8.27% and 4.52%, respectively, and improves the average cost by 21.56%.

## 1. Introduction

The Internet of Things (IoT) is a network that connects various pervasive objects, such as smartphones, sensors, actuators, and Radio Frequency Identification (RFID) tags, through the Internet [1,2,3,4]. It bridges the gap between the physical and virtual worlds, becoming essential to everyday life [5,6,7]. This dynamic network infrastructure possesses self-configuring capabilities facilitated by standard and interoperable communication protocols [8,9,10,11,12]. To enable connectivity, various communication technologies are employed in this field, including Near-Field Communication (NFC), Wireless Sensor Networks (WSN), Long-Term Evolution (LTE), Radio Frequency Identification (RFID), and others [13,14,15,16]. Components like wireless sensor networks, RFID, data acquisition, actuator networks, and managerial control are integral aspects of the IoT [17]. The connected objects collect data and share information about their operations [18,19]. Advancements in Information Technology (IT) have led to rapid IoT development, allowing its implementation in diverse fields such as agriculture, industry, the military, home monitoring, and more [20,21].

Cloud computing has revolutionized access to vast resources, offering easy and instant availability [22,23,24]. Combined with the IoT, this integration enhances the virtual resource infrastructure and available services [25,26,27,28]. By merging the cloud and IoT, both platforms can mutually benefit. On the one hand, IoT can leverage the cloud’s extensive capabilities, addressing its technological limitations in storage, processing, and communication. The cloud’s limitless potential compensates for these shortcomings. IoT enables the cloud to dynamically access real-world objects and extend its application to a broader range of scenarios [29]. Moreover, the emergence of fog computing has addressed the need to process IoT data in edge networks closer to IoT sensors [30,31,32,33,34]. This approach reduces inherent delays in cloud data centers and facilitates the discovery of improved services [35].

Huge amounts of data are passed over the Internet every day [36]; therefore, we need to find a way to merge these data and services to meet the needs of customers. Service composition in the IoT can benefit from both fog and cloud computing, each with its own advantages and disadvantages. Fog computing, a decentralized architecture that enables computation closer to the data source, reduces latency, and improves overall performance [37,38]. It is particularly suitable for real-time applications like video analytics and those requiring high levels of security and privacy [39,40]. On the other hand, cloud computing, a centralized architecture reliant on remote servers, is useful for applications with extensive storage and processing requirements without immediate response times. Therefore, the choice between fog and cloud computing for IoT service composition hinges on the application’s specific needs. If low latency, high security, and real-time processing are crucial, fog computing becomes the better choice. However, cloud computing is more suitable when extensive storage, processing power, and scalability are required. In some instances, a combination of both fog and cloud computing may provide the optimal solution to balance performance, security, and scalability requirements. IoT devices typically exhibit heterogeneous and dynamic embedded characteristics, with each device responsible for individual atomic services [41]. With the growing number of connected IoT objects, the selection of web services becomes crucial in addressing user requests [42,43]. In cases where a single service cannot handle user requests, multiple services need to be composed to operate IoT-based system applications [44,45]. Therefore, integrating services provided by IoT devices is necessary. Energy consumption is a critical concern, particularly because most IoT smart devices rely on batteries for power [46,47]. Combining IoT and composite services is essential to ensuring battery longevity and meeting user requirements while optimizing energy consumption. In service composition, replacing energy-intensive intelligent devices with more efficient alternatives that offer the same functionality and QoS level is essential. Selecting the best web services to create an optimal composite service is a challenging NP-complete problem [48,49]. A fuzzy-based hybrid algorithm combines ACO and ABC methods to address this. This hybrid approach evaluates QoS parameter values and energy consumption to achieve an optimal solution for IoT service composition. The proposed algorithm models IoT service composition as a multi-objective optimization problem with the primary goal of optimizing QoS parameters for the composite service. To narrow down the solution search domain, candidate services are pre-selected to meet users’ QoS requirements and determine the optimal composite service. The service composition problem is then transformed into finding an optimal path with specific QoS requirements within a directed acyclic graph, representing possible service connections and interactions. To find the best solution for the service composition problem, the hybrid algorithm combines the strengths of ACO and ABC. These two algorithms provide different approaches for solving similar problems, and the hybrid approach aims to leverage their advantages to achieve an optimal solution. The optimization process focuses on simultaneously reducing energy consumption and optimizing QoS parameters. The primary objective is twofold: first, to minimize energy consumption by reducing the resources utilized during the execution of composed services; and second, to optimize QoS parameters such as reliability, response time, and other performance metrics. By addressing both aspects, the algorithm seeks to create efficient and high-performing composite services for IoT applications. The optimization process of the developed IoT service composition methods takes into account both energy consumption and QoS parameters simultaneously, ensuring an efficient and high-quality composition of services.

The main contributions and novelties of the developed IoT service composition methods can be summarized as follows:

1. Integration of cloud, fog, and IoT: The proposed methods aim to integrate cloud computing, fog computing, and IoT technologies to leverage their respective advantages. This integration enhances the virtual resource infrastructure and available services. By combining these technologies, IoT devices can benefit from the unlimited resources and capabilities of the cloud while reducing latency and processing data at the edge networks through fog computing. The novelty lies in combining these technologies to create a more efficient and scalable service composition approach.
2. Fuzzy-based hybrid algorithm: The developed IoT service composition methods employ a fuzzy-based hybrid algorithm, which is a novel approach in the field. This algorithm combines the ACO and ABC algorithms. The methods can handle the uncertainty and imprecision inherent in IoT environments by integrating fuzzy logic into the algorithm. The fuzzy-based hybrid algorithm considers multiple QoS parameters simultaneously, leading to improved optimization and selection of services.

The organization of the rest of the paper is as follows: Section 2 provides an explanation of related works in the field. In Section 3, the proposed method is presented. Section 4 covers the experiment results. Finally, Section 5 discusses the conclusion and future work.

## 2. Related Work

In this section, we review some recent studies investigating service composition mechanisms in the context of IoT.

Asghari, Rahmani [41] proposed a privacy-aware service composition technique. They employed a hybrid evolutionary algorithm and an IoT-based conceptual model to optimize various Quality of Service (QoS) parameters in the service composition process. The hybrid algorithm, SFLA-GA, combines a Genetic Algorithm (GA) and a Shuffled Frog Leaping Algorithm (SFLA). The fitness value, representing the aggregation of different QoS factors, is optimized using this method. Simulation results showed that SFLA-GA outperformed other contemporary algorithms. One of the strengths of this method is its focus on privacy awareness in cloud-IoT service composition as well as QoS optimization. However, it lacks consideration for energy, security, and lightweight encryption.

Sefati and Navimipour [50] proposed an approach to optimize Quality of Service (QoS) for service composition in the IoT. They combined an Ant Colony Optimization (ACO) algorithm with a hidden Markov model to address the service composition issue. The method employed Markov clustering based on differentiating operators, which were repeatedly applied to the network to identify clusters. This process involves increasing edge values within clusters while decreasing edge values between clusters. These operators are faster, simpler, and more natural for clustering, making them suitable for various applications. The evaluation of QoS in this research was accomplished via the ACO algorithms, which helped to find an appropriate path. The primary focus of this approach was on achieving high reliability and availability. However, energy efficiency was not a major consideration in their work.

Souri and Ghobaei-Arani [51] proposed a service composition method specifically designed for cloud manufacturing and Industrial IoT applications. The significance of this approach lies in its applicability to industrial settings. The method utilized a formal verification strategy based on a Labeled Transition System (LTS) to evaluate the proposed solution. Moreover, the Whale Optimization Algorithm (WOA) was employed to enhance Quality of Service (QoS) for cloud services. The verification process focused on ensuring reachability factors, deadlock-free conditions, verification time, and memory usage based on different cloud providers and various Linear Temporal Logic (LTL) properties. Despite the achievements in formal verification and QoS improvement using WOA, the proposed algorithm had some drawbacks. The WOA exhibited slow convergence, a tendency towards local optima, and lower accuracy compared to other optimization algorithms. Additionally, the energy consumption aspect was not adequately addressed in their approach. This service composition method for cloud manufacturing and IoT applications is notable for its industrial relevance and formal verification approach. However, improvements are needed to address the WOA’s limitations and include energy consumption considerations in the optimization process.

Moreover, Chai, Du [46] presented an algorithm for IoT service composition that aimed to achieve better QoS while considering energy efficiency. The proposed algorithm combined QoS-aware service composition with a fast energy-centered algorithm, FSCA-EQ, utilizing a hierarchical optimization method. First, the algorithm selected candidate services with better QoS using a Customer Relationship Management (CRM) approach. Subsequently, the relative dominance concept was employed to determine the highest-quality service from the pool of candidate services. The results demonstrated that the proposed method achieved better energy consumption, selection time, and overall optimality. However, a limitation of this approach was that it did not account for the dynamic characteristics of the IoT. As a consequence, the service selection process for energy efficiency needed to be rerun frequently, resulting in a high computational cost. The proposed algorithm combined QoS-awareness and energy optimization effectively. Nevertheless, the lack of consideration for the IoT’s dynamic nature led to increased computational expenses due to frequent service selection reruns.

Ibrahim, Rashid [52] proposed an energy-aware mechanism to enhance mobile cloud service composition. The approach utilized a hybrid algorithm based on the Shuffled Frog Leaping Algorithm (SFLA) and Genetic Algorithm (GA), called SFGA, to optimize service composition based on multiple quality measurement parameters: cost, energy, and response time. By employing the SFGA algorithm, service selection was performed faster, and the overall service composition process was carried out more effectively regarding service cost and response time. The proposed method outperformed existing approaches, increasing the likelihood of service composition with minimal reaction time, energy consumption, and cost for mobile cloud components, as evidenced by testing results. However, it is worth noting that this approach did not consider other important IoT parameters, such as reliability and availability. While the method excelled in energy-aware service composition, it did not address other critical aspects that are essential in IoT applications.

Jian, Li [53] proposed a strategy for optimizing QoS-based service composition in edge computing. They combined Multi-Objective Particle Swarm Optimization (MPSO) and the Bird Swarm Algorithm (BSA) to enrich the search information throughout the optimization process and avoid getting trapped in local optima. The results demonstrated that their proposed method outperformed other papers considered in the research in terms of providing a better solution for achieving the global optimal QoS value for edge services. The algorithm ensured high reliability and low execution time, making it a strong choice for QoS-based service composition in edge computing applications. However, it is important to note that the algorithm did not consider energy consumption and availability during optimization. While the proposed method focused on QoS optimization, it did not address energy efficiency and service availability, which are crucial considerations for edge computing and IoT applications.

Guzel and Ozdemir [45] proposed a multi-objective IoT service composition framework designed for fog-based IoT environments. This framework aims to generate service composition strategies that consider Quality of Service (QoS), energy consumption, and fairness. The task is treated as a multi-objective optimization problem to obtain effective composition techniques for IoT applications. A generic QoS model was developed and integrated into the framework to handle the diversity and ever-changing characteristics of the IoT domain. The application requests were divided into time windows, and each window was optimized using the defined optimization model. The optimization method focused on reducing energy consumption, minimizing the repeated use of the same IoT services, and minimizing the number of broken Service Level Agreements (SLAs). The test findings showed a trade-off between energy consumption and fairness goals. The architecture tended to prioritize IoT services with low energy consumption, driven by the energy consumption goal. However, this approach did not consider scalability, which is a crucial aspect for IoT systems that may need to accommodate a growing number of devices and users. In summary, Guzel and Ozdemir’s multi-objective IoT service composition framework for fog-based IoT environments provides composition strategies considering QoS, energy consumption, and fairness. The use of a generic QoS model allows adaptability to diverse IoT characteristics. However, the framework does not address scalability, which could be a limitation in scenarios with increasing IoT device and user numbers.

Naseri and Navimipour [54] introduced a hybrid method that combines an agent-based approach with the Particle Swarm Optimization (PSO) algorithm for efficient service composition in cloud computing. The method identifies relevant Quality of Service (QoS) parameters and selects the best services based on a fitness function. The results demonstrated the effectiveness of this approach in reducing resource usage and waiting time and optimizing resource allocation via the efficient composition of independent services into composite services. This can lead to cost savings and improved resource utilization. The hybrid PSO algorithm showcased promising results compared to other algorithms, suggesting its efficiency in finding suitable composite services. This can ultimately enhance performance and service quality in cloud computing environments. However, it is worth noting that the agent-based method used for service composition might lack a clear mechanism for combining resources. The PSO algorithm was incorporated to address this limitation, which may introduce additional complexity to the overall system. The proposed hybrid method for cloud service composition effectively combines an agent-based approach with the PSO algorithm to optimize resource usage and waiting time. Although it enhances the composition process, integrating the PSO algorithm may add complexity to the system. The proposed method demonstrates promising results in finding the right composite services for cloud computing environments.

Chen, Wang [55] introduced an optimal objective for web service composition selection, incorporating the concept of QoS satisfaction degree. The authors proposed the use of a genetic algorithm to solve this problem and provided test results that demonstrated the feasibility and effectiveness of their proposed solution. By considering the subjective feelings of users, the proposed model aimed to enhance users’ satisfaction and experience during web service composition selection. The utilization of a genetic algorithm in their approach offers a robust and efficient method to address the multi-objective optimization problem, potentially leading to improved solutions. Their research primarily focused on QoS satisfaction as the main objective for web service composition selection. It is important to note that the effectiveness and feasibility of the proposed solution may vary depending on the specific context and the range of services available. Different scenarios and diverse sets of web services may influence the results. 

Ullah, Ali [56] proposed an energy optimization strategy for the smart grid integrated with renewable energy sources to minimize operational costs and carbon emissions. To handle the uncertainty of solar and wind energy sources, probability density functions are used to predict their behavior. Demand response programs involving residential, commercial, and industrial consumers were introduced with incentive-based payment packages to optimize energy consumption. The optimization model employed a Multi-Objective Genetic Algorithm (MOGA) to solve the energy optimization problem. The simulations compared the proposed MOGA-based model with an existing one that utilized the Multi-Objective Particle Swarm Optimization algorithm, showing better performance in reducing operational costs and carbon emissions. The proposed strategy effectively integrated solar and wind energy sources into the smart grid, enabling better utilization of renewable energy and reducing dependence on fossil fuels. The use of probability density functions for predicting the behavior of solar and wind energy sources helped handle the inherent uncertainty in renewable energy generation, leading to more accurate and reliable optimization results. The proposed energy optimization strategy based on MOGA demonstrates the potential to optimize smart grids’ operational costs and carbon emissions when integrated with renewable energy sources. Addressing computational complexity and conducting real-world validation will be essential for practical deployment and ensuring the model’s effectiveness under diverse operating conditions.

Ullah, Khan [57] proposed a new Demand-Side Management (DSM) strategy for the day-ahead scheduling problem in smart grids with high wind energy integration. The strategy aimed to optimize a tri-objective problem by minimizing operating costs, pollution emissions, and load curtailment costs while ensuring coordination between wind turbine output power and demand. The model used the Monte Carlo simulation for wind energy prediction to account for the uncertainty of wind energy. The DSM strategy involved real-time pricing and incentives, forming a hybrid demand response program (H-DRP). The optimization technique used to solve the tri-objective smart grid scheduling problem was a multi-objective genetic algorithm that incorporated a decision-making mechanism to find the optimal solution. The simulation results demonstrated the successful optimization of the objective functions using the proposed model. The integrated DSM strategy, distributed energy resources, and H-DRPs lead to environmental benefits by minimizing pollution emissions and economic benefits by reducing operating costs.

Ali, Ullah [58] introduced the demand-side management (DSM) strategy in a smart grid to optimize energy management by involving distributed energy resources and considering different types of consumers. The day-ahead scheduling problem was solved using a multi-objective wind-driven optimization algorithm to optimize operational cost, pollution emissions, load curtailment cost, and coordination between wind energy generation and shiftable loads. The model also considered consumer behavior and the probabilistic nature of wind energy forecasting using probability distribution functions. Simulation results showed that the proposed model effectively reduced operational costs and pollution emissions. Through day-ahead scheduling with multi-objective optimization, the DSM strategy enables efficient energy management by simultaneously considering various objectives. The model’s implementation reduced operational costs for the utility grid, particularly when involving distributed energy resources such as wind turbines, diesel generators, and energy storage systems. Including multi-objective optimization and probabilistic modeling makes the proposed approach more complex to implement and understand, potentially requiring specialized expertise. Overall, the advantages of the proposed DSM-based multi-objective day-ahead scheduling model outweighed the potential disadvantages. However, further research, testing, and real-world implementation are necessary to fully validate its applicability and performance in different smart grid scenarios.

Hafeez, Wadud [59] focused on meeting the increasing electrical energy demand in IoT-enabled residential buildings and proposed an energy management strategy using the Wind-driven Bacterial Foraging Algorithm (WBFA). The strategy aimed to optimize the power usage of smart appliances and participate in demand response (DR) programs to manage energy consumption efficiently. Simulations demonstrated that the WBFA-based strategy outperformed benchmark algorithms regarding energy consumption, cost of electricity, peak-to-average ratio, and user comfort. The study concluded that employing price-based DR programs, particularly the Time-of-Use Price-based DR program, yields favorable outcomes for consumers and Distribution System Operators. Future research directions include extending energy management to coordinate with power grids, renewable energy sources, energy storage, and electric vehicles (EVs) for prosumers (consumers who generate and sell energy). The study suggested using machine learning to explore fog and cloud-based energy management, hybrid generation systems, and intelligent forecasting, additionally proposing innovative energy management models for cloud computing and integrating time- and power-flexible appliances to provide economical and sustainable solutions. The WBFA-based strategy optimized the power usage of smart appliances, leading to increased energy efficiency and effective energy utilization in IoT-enabled residential buildings. By participating in demand response (DR) programs and scheduling power usage during off-peak hours, consumers can benefit from lower electricity costs, saving money on their utility bills. The proposed WBFA-based energy management strategy offered several advantages, including energy efficiency, cost reduction, and peak load management. However, there are challenges, such as complexity, compatibility issues, and potential reliability concerns. Careful planning, education, and addressing technological limitations are essential to maximizing the benefits and overcoming the disadvantages of this approach.

A summary of the related works is presented in Table 1.

## 3. Proposed Method

This section outlines the proposed method for solving the service composition problem in the context of IoT. Section 3.1 presents the system model; Section 3.2 is about the service composition model; Section 3.3 is about the energy model; and Section 3.4 is about fuzzification. In Section 3.5, a hybrid algorithm is presented.

### 3.1. System Model

**Figure 1:**
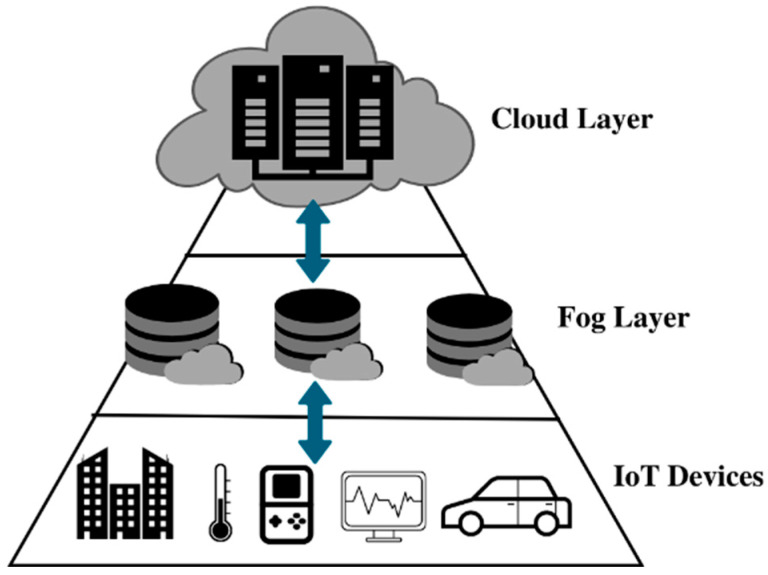
System Architecture.

This section introduces the proposed composition strategy for a fog-/cloud-based IoT environment, where the architecture combines the advantages of fog and cloud computing to create a more efficient data handling system. The architecture is designed such that fog computing operates at the network’s edge, enabling real-time data processing and analysis. In contrast, cloud computing is utilized for storing and analyzing large volumes of data over an extended period. The fog layer, comprising small, low-power devices located near the data source, is responsible for real-time data processing and analysis. These devices can be programmed to take specific actions based on the results of their analysis. On the other hand, the cloud layer consists of larger, more powerful servers typically situated in centralized data centers, handling more complex data processing tasks such as machine learning and big data analytics. The combination of fog and cloud computing offers various benefits, including reduced latency and improved response times through real-time processing at the fog layer and the ability to handle more resource-intensive tasks using the cloud layer’s storage and processing capabilities. This architecture enhances data processing and analysis, improving organizations’ performance, efficiency, and scalability. Moreover, it also leads to cost reduction and enhanced security. The proposed technique adopts a three-tier architecture comprising IoT, fog, and cloud layers (Figure 1) [60,61]. The IoT layer encompasses sensors and smart nodes, like computers and cell phones, forming the Internet of Things layer. The fog layer between the cloud and the IoT smart nodes contains fog colonies. Sensors at the lower layer collect diverse information and send data packets to the fog layer. Here, fog nodes intelligently accept and process all requests. Depending on whether they rely on real-time applications, queries are either handled within the fog layer or sent to the top layer, the cloud layer. By adopting this three-tier architecture, the proposed technique aims to optimize data processing and analysis within the IoT environment, promoting efficient decision-making and intelligent service composition.

### 3.2. Service Composition Model

**Figure 2:**
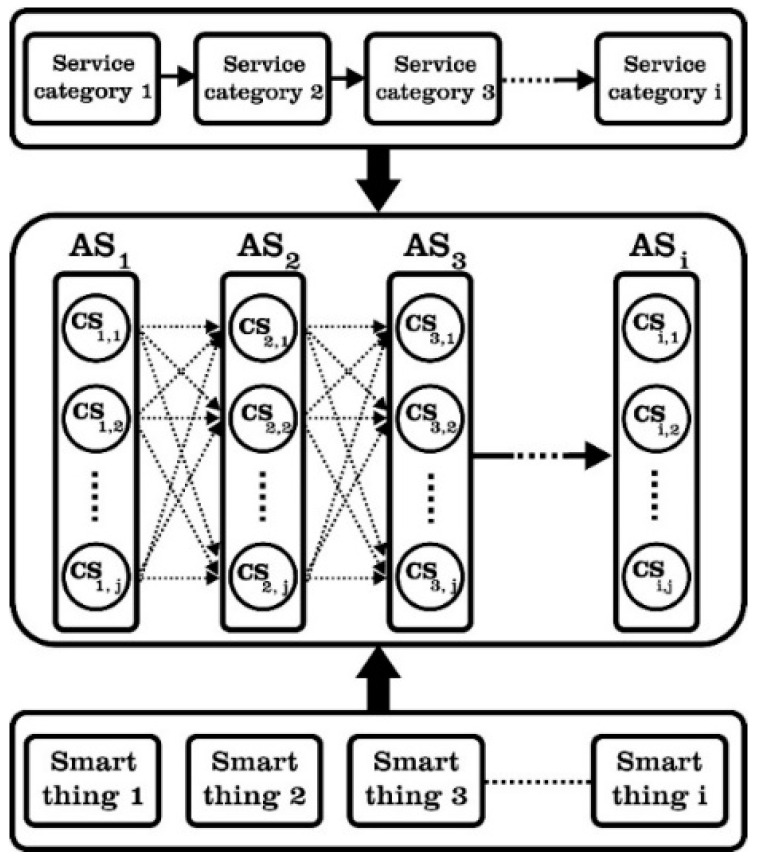
The QoS composition process of IoT.

In the context of the IoT, IoT nodes serve as encapsulated IoT services and are categorized into specific service classes based on their functionalities [62]. The services in the IoT service composition fall into two distinct categories: Concrete services (Cs) and Abstract services (As) [46]. As is also called an abstract service class. Cs, or atomic services, represent invocable services provided by IoT components. Cs can be described by two attributes: non-functional features and functional characteristics. Functional attributes pertain to the specific functions offered by a service, while non-functional attributes encompass quality-of-service (QoS) aspects such as response time, reliability, cost, and energy profiles for services operating on battery-powered devices, among others. Figure 2 illustrates the fundamental process of IoT service composition [46]. In a composite service, atomic services can be interconnected using various structural patterns. There are six types of composition structure patterns that a single composite service can comprise: sequential, AND split (Fork), XOR split (Conditional), Loop, AND join (Merge), and XOR join (Trigger) [63,64]. Here, only the sequential model is considered. However, it is worth noting that other models can be simplified or transformed into sequential models using existing approaches, as mentioned in [65].

In the IoT, each object has the capability to offer multiple specific services. Some IoT services may provide the same functionality but exhibit different Quality of Service (QoS) performance levels. Therefore, evaluating QoS parameters becomes essential to differentiate between services. In this paper, service composition is approached at a high level, considering the sequence of services as a workflow. QoS in the context of IoT services encompasses various non-functional characteristics of an application, such as throughput, response time, availability, security, and reliability. Service providers may offer some QoS values while the users determine others. QoS is user-dependent, meaning different users may have varying requirements for attributes like availability, response time, reliability, resource costs, packet loss rate, etc. For the assessment of services, four QoS properties have been selected in this paper:

(1) Availability: This represents the percentage of time a service is accessible during a specific time interval.
(2) Reliability: Refers to the percentage of a service’s capability to perform correctly without errors or failures.
(3) Cost: This denotes the price a user pays to obtain the required service.
(4) Energy: This represents the energy a service consumes during its operation.

As Table 2 outlines, the paper employs specific QoS aggregation functions for each attribute to evaluate the proposed dynamic service composition scheme. By applying these QoS values, the scheme can effectively determine the optimal service composition based on users’ requirements and preferences, taking into account factors like availability, reliability, cost, and energy consumption.

Since there is a major distinction in the QoS index range and estimation unit, combining diverse QoS indexes directly when the objective function is calculated is impossible. In order to map the four aggregated QoS right into a global value, the Simple Additive Weighting (SAW) approach is used. Given that the minimization objective function is considered in this paper, the positive and negative normalization formulations are shown in (1) and (2), respectively [54]. In the two equations below, the *i*th attribute value of the concrete service is represented by *Cs.Qi*. The highest and the lowest values of the *i*th attribute among all the concrete services in the service candidate set are represented by $Q_{max}^{i}$ and $Q_{min}^{i}$ respectively.

(1) $$N_{Cs.Q^{i}}=\{\begin{array}{c} \frac{Q_{max}^{i}-cs.Q^{i}}{Q_{max}^{i}-Q_{min}^{i}}\\ 1 \end{array}\begin{array}{c} \;Q_{max}^{i}\neq Q_{min}^{i}\\ \;Q_{max}^{i}=Q_{min}^{i} \end{array}$$

(2) $$N_{Cs.Q^{i}}=\{\begin{array}{c} \frac{cs.Q^{i}-Q_{min}^{i}\;}{Q_{max}^{i}-Q_{min}^{i}}\\ 1 \end{array}\begin{array}{c} \;Q_{max}^{i}\neq Q_{min}^{i}\\ \;Q_{max}^{i}=Q_{min}^{i} \end{array}$$

The fitness function influences the algorithm’s convergence and the search for an optimal solution. The fitness of an individual solution is calculated using the formula below [66]:

(3) $$\mathrm{Fitness}=\sum_{i=1}^{4}W_{i}*Q_{i}\;$$

*W_i_* indicates the weight value of the *i*th QoS attribute of one atomic service, 0≤W≤1, $\overset{4}{\underset{i=1}{\sum }}W_{i}=1$. *Q_i_* indicates the *i*th QoS aggregation attribute value of the solution.

### 3.3. Energy Model

The amount of energy consumed in IoT service composition greatly affects devices hosting the candidate service. Each candidate service must possess a parameter representing its energy consumption. This parameter allows the composer to select services with a better energy-saving effect. The energy profile of concrete service *EproFile(Cs_ij_)* is a composition of the following variables [46]:

The autonomy of the service *SA(Cs_ij_)* is defined as the energy level of the device that hosts this service:

SA(*Cs_ij_*) = CE(Cs_ij_) − ET(Cs_ij_) (4)

In this equation, *CE(Cs_ij_)* describes the current energy level of the battery-powered device that hosts service *Cs_ij_. ET(Cs_ij_)* means the energy threshold of the battery-powered device that can host service *Cs_ij_.*

The energy consumption *EC(Cs_ij_)* for running a concrete service *Cs_ij_* is constant and can be calculated according to the equation below:

EC(Cs_ij_) = ECR(Cs_ij_) × RT(Cs_ij_) (5)

In this equation, *RT(Cs_ij_)* means the average running time of service *Cs_ij_*, and *ECR(Cs_ij_)* means the energy consumption rate.

Therefore, the following equation is proposed for the energy profile *EproFile(Cs_ij_)* in the service *Cs_ij_.*

(6) $$EProFi({Cs}_{ij})=\frac{EC\left({Cs}_{ij}\right)}{SA\left({Cs}_{ij}\right)}$$

A low amount of energy profile means that the IoT device hosting the service *Cs_ij_* has a relatively long service life. Consequently, the following equation is proposed for the energy profile of composite services *CEProFile(x):*

(7) $$CEProFi(x)=\sum_{i=1}^{n}EProFile(x^{i})$$

In this equation, *x^i^* is the *i*th component in the composite service selected from the abstract service class.

### 3.4. Fuzzification

**Figure 3:**
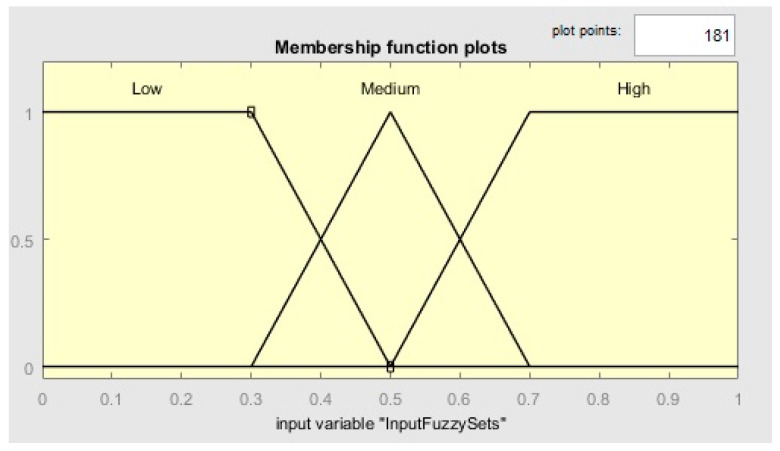
Input fuzzy sets (availability, reliability, cost, and energy).

**Figure 4:**
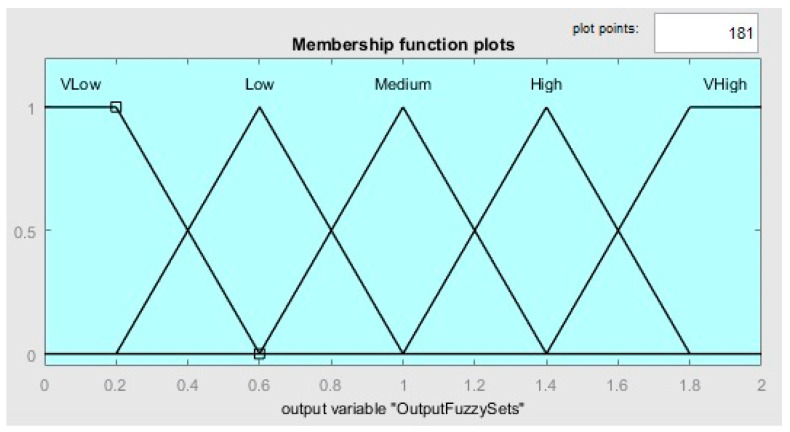
Output fuzzy sets (pheromone).

Many industries and scholars utilize fuzzy logic since it can operate in artificial systems when automated decision-making is required [67]. Mamdani and Assilian [68] have introduced the fuzzy logic controller, which is currently considered one of the most critical applications of fuzzy set theory [69]. Some of its relevant characteristics are fuzzy rules, linguistic variables, and fuzzy sets. A fuzzy set is a group of objects characterized by a membership function ranging between 0 and 1 [70]. In this process, the variable type utilized is words rather than numbers called linguistic variables (e.g., fast, medium, and slow) [71]. The values employed to describe linguistic variables are called terms, and their collection is called a term set. Fuzzy rules (IF-THEN) are utilized to evaluate the situations. Following the fuzzification phase, crisp inputs are converted to linguistic variables, the fuzzy rules assess them, and the outputs are created. The linguistic values from the outputs are converted to crisp values during the defuzzification stage. The fuzzy inference engine proposed in this paper has one output and four inputs. All inputs are described according to the same membership functions in Figure 3. These inputs are normalized availability, reliability, cost, and energy. In order to define the high and low terms, trapezoidal-shaped functions are utilized, whereas triangular-shaped functions are employed for the medium term, as can be seen in Figure 3. The Mamdani inference system with a centroid of area defuzzification strategy is employed for our fuzzy engine. In this strategy, the pheromone for the ant is determined by the output value of the fuzzy inference system (Figure 4). IF-THEN fuzzy rules employ fuzzy sets to create output in the inference system. A fuzzy system can play a significant role in service composition in the IoT by providing intelligent decision-making capabilities. Fuzzy systems in service composition in the IoT enable handling uncertainty, incorporate context-awareness, support decision-making processes, and facilitate service matching and optimization. By leveraging fuzzy logic techniques, IoT systems can dynamically and intelligently compose services to meet specific requirements and adapt to changing conditions flexibly and robustly. The advantages of using a fuzzy-based hybrid algorithm that combines the ABC and ACO algorithms in service composition in IoT include handling uncertainty, flexible fitness evaluation, dynamic parameter tuning, context-aware decision-making, improved optimization, and enhanced performance and scalability. These advantages enable the algorithm to generate more robust, adaptive, and high-quality service compositions, aligning them with IoT environments’ diverse requirements and dynamic nature. Some advantages of using a fuzzy-based hybrid algorithm that combines the ABC and ACO algorithms for service composition in IoT:

- Handling Uncertainty: IoT environments often involve uncertain and imprecise information due to varying data quality, incomplete knowledge, and ambiguous conditions. Fuzzy logic, employed in the hybrid algorithm, allows for representing and reasoning with imprecise and uncertain data. It enables the algorithm to handle uncertainty in evaluating service quality attributes, decision-making, and optimization, resulting in more robust and adaptable service compositions.
- Flexible Fitness Evaluation: The fuzzy-based hybrid algorithm allows flexible fitness evaluation by considering multiple quality attributes simultaneously. The fuzzy system, integrated into the hybrid algorithm, utilizes membership functions and fuzzy rules to evaluate the suitability and relevance of IoT services based on attributes such as availability, reliability, cost, and energy. This comprehensive evaluation leads to more accurate fitness assessments by considering various factors relevant to service composition in the IoT.
- Dynamic Parameter Tuning: The hybrid algorithm combines the strengths of the ABC and ACO algorithms. The fuzzy system enhances this combination by facilitating dynamic parameter tuning based on the problem context and performance feedback. Fuzzy rules are employed to adjust algorithm parameters such as pheromone evaporation rate, colony size, and exploration-exploitation balance. This adaptability enables the algorithm to navigate the search space effectively and optimize the service composition process.
- Context-Aware Decision-Making: The fuzzy system integrated into the hybrid algorithm enables context-aware decision-making. The algorithm can adapt its decision-making process by considering contextual factors such as user preferences, environmental conditions, and resource constraints. Fuzzy inference mechanisms assess the importance and relevance of different decision criteria, allowing for more intelligent and personalized service composition in IoT scenarios.
- Improved Optimization: The hybrid algorithm, with the fuzzy system, provides enhanced optimization capabilities. Fuzzy optimization techniques can be employed to refine and optimize the service compositions generated by the algorithm. These techniques explore different combinations of services, adjust parameters, and optimize resource allocations to achieve near-optimal solutions. The hybrid algorithm can effectively balance trade-offs, handle constraints, and consider user preferences during optimization by leveraging fuzzy logic.
- Enhanced Performance and Scalability: The combination of the ABC and ACO algorithms, empowered by the fuzzy system, can lead to improved performance and scalability in service composition for IoT. The fuzzy-based hybrid algorithm allows for efficient exploration of the search space, exploitation of the best solutions, and dynamic adaptation to changing conditions. It can handle complex and large-scale IoT environments effectively, providing more efficient and scalable service compositions.

### 3.5. Hybrid Algorithm

In 2005, Abd-Alsabour and Randall [72] introduced the so-called ABC, an intelligent swarm algorithm. There are three types of bees in the colony. They are Onlooker Bees (OBees), Employed Bees (EBees), and scout bees. At first, the food source positions are created (*N*). Each food source is devoted to an employed bee. Having exploited the food sources, EBees send the nectar amount information to OBees. Subsequently, OBees initiate the exploitation of food sources, giving priority to those with higher quality. When a source is depleted, scouts start to search for a new food source. However, the nectar information is, in fact, the quality of the available food source solution. The number of food sources and the population of EBees and OBees are equal.

In the early 1990s, Dorigo and his colleagues proposed ACO [73]. This algorithm consists of real ants’ coordinated behavior and their self-organizing principles to find a solution for complex combinatorial optimization problems. Ants are considered social insects that can find the shortest route between the nest and the food source through pheromone updates, evaporation, and path construction. The ABC algorithm and the ACO algorithm are both metaheuristic optimization algorithms that can be used to solve a wide range of complex problems. Here are some disadvantages of both algorithms:

- Slow convergence: ABC and ACO algorithms can converge slowly, especially when dealing with complex problems with a large search space. This can result in longer optimization times and may be unacceptable in some applications.
- Premature convergence: Both algorithms can suffer from premature convergence, where the algorithm gets stuck in a local optimum and cannot find the global optimum. This can result in suboptimal solutions and may require additional optimization runs to obtain better solutions.
- Parameter sensitivity: The ABC and ACO algorithms have several parameters that need to be set, and their values can significantly affect the algorithm’s performance. Setting these parameters can be challenging, and incorrect values can lead to poor optimization results.
- Inability to handle constraints: Both algorithms are not well-suited for problems with constraints, as they do not provide an explicit mechanism for handling them. This can result in infeasible solutions, which may not be useful in some applications.
- Limited memory: Both algorithms do not store the previous search history, which can limit their ability to explore the search space efficiently. This can result in inefficient searches and longer optimization times.

Hybrid algorithms combine two or more optimization techniques to enhance the algorithm’s overall performance. ACO and ABC are two optimization techniques successfully used in solving various optimization problems. Here are some advantages of using a hybrid algorithm:

- Improved global search: The hybrid approach can combine the strengths of both the ACO and ABC algorithms to improve global search. ACO is good at exploring the search space, while ABC exploits good solutions. By combining the two approaches, the hybrid algorithm can better balance exploration and exploitation, resulting in better optimization results.
- Faster convergence: The hybrid algorithm can converge faster than the individual algorithms since it can take advantage of the strengths of both algorithms. This can result in shorter optimization times, which can be important in many applications.
- Robustness: The hybrid algorithm can be more robust than the individual algorithms since it can handle a wider range of optimization problems. This is because the hybrid algorithm can adapt to different problem characteristics, taking advantage of the strengths of both the ACO and ABC algorithms.
- Better handling of constraints: The hybrid algorithm can handle constraints better than individual algorithms since ACO has a mechanism for handling constraints. This can result in more feasible solutions, which can be important in many applications.
- Flexibility: The hybrid algorithm can be easily customized to suit different optimization problems by adjusting the parameters of the individual algorithms. This can make it a more versatile approach for solving different optimization problems.

Due to too many intensifications by the OBees, ABC’s convergence is accompanied by a delay, and on the other hand, ACO during intensification has stagnation. Ants create the initial set of solutions, and the routes are handed over to bees for exploitation to tackle these issues in the proposed AC-ABC hybrid algorithm. The advantages of the ACO and ABC algorithms are hybridized and synergized in the proposed algorithm. In the proposed algorithm, ants create paths that combine optimal services. These services are, in fact, the initial food sources for the bee colony. When sources are given to ABC, the EBees search the services, their neighborhoods, and the information sent to the OBees. The OBees tend to be the food source with higher quality (fitness), so solutions with higher fitness will have more chances of being selected by the OBees. Then the scout bees replace the exhausted food sources with new, random ones. Bee-optimized food sources are given to ants as their path to updating pheromones based on a fuzzy inference system. In the next iteration, ants will tend to follow paths with more pheromones. Finally, the algorithm will halt when it reaches a predetermined number of iterations. The comprehensive explanation of the suggested algorithm and the equations included are considered below:

Step 1: Initialize the algorithm parameters. In this step, the necessary parameters for the algorithm are set, such as the number of ants, the number of iterations, the evaporation rate of pheromones, and any other parameters specific to the problem being solved (Number of iterations: 100, number of ants: 20, α = 0.6, β = 0.4, evaporation rate of pheromones: 0.2).

Step 2: Normalize QoS Values. The QoS values, which represent the performance attributes of candidate services, are normalized to a common scale. This step ensures that the QoS values can be compared and combined effectively.

Step 3: The *Q_score_* is calculated for each of the candidate services for each task [66]. *Q_score_* is a measure of the quality of a candidate service based on its QoS attributes. The *Q_score_* is calculated using a weighted sum of the QoS attribute values, multiplying each attribute by a corresponding weight. The weights determine the relative importance of each attribute.

(8) $$Q_{\mathrm{score}}=\sum_{i=1}^{4}W_{i}*Q_{i}$$

*W_i_* denotes the weight value of the *i*th QoS attribute of one atomic service, $0\leq W_{i\;}\leq 1$, $\overset{4}{\underset{i=1}{\sum }}W_{i}=1$. *Q_i_* denotes the *i*th QoS attribute value. Four in (8) is considered as the number of the parameters that should be optimized.

Step 4: The probability of selecting *ij* path by ant *k* is calculated due to the pheromone and service’s score [74]. The probability of selecting a particular path (combination of services) by an ant is calculated based on the pheromone level on the path and the *Q_score_* of the services in the path. The pheromone level determines the attractiveness of the path, while the *Q_score_* reflects the quality of the services. The influence of pheromones and *Q_score_* is controlled by positive parameters, alpha (α) and beta (β), respectively.

(9) $$P_{ij}^{k}=\{\begin{array}{c} \frac{\tau_{ij}^{\alpha }\;Q_{score\;ij}^{\beta }}{\sum_{m\epsilon N_{i}^{k}}{\;\tau }_{im}^{\alpha }\;Q_{score\;im}^{\beta }}\;j\epsilon N_{i}^{k}\\ 0\;otherwise \end{array}$$

where *α* and *β* are positive numbers to determine the influence of pheromones and quality of the candidate services, respectively. $\tau_{ij}$ is the amount of pheromone on path *ij*. $N_{i}^{k}$ is the set of possible neighborhoods that ant *k* could pass through them.

Step 5: The probability values of services are entered into a roulette wheel selection, and a service is selected for each task. The probability values calculated in the previous step are used to perform roulette wheel selection. Each ant selects a service for each task based on these probabilities. This selection process ensures that services with higher probabilities (higher pheromones and *Q_score_*) are more likely to be selected.

Step 6: The paths created by the ants are given to the EBees as the initial solutions. The paths created by the ants, which represent solutions, are passed to the EBees as their initial solutions. The EBees will further explore and improve these solutions.

Step 7: EBees receive solutions and start to look for a neighboring food source by “neighborhood search” or “local search” based on Equation (10) [75,76]. The EBees started to carry out a local search to improve the solutions they received. They perform a neighborhood search or local search for each task. This search involves evaluating the QoS parameters of the selected service and creating a virtual service based on these parameters. The most similar service to the virtual service in the same task is found using the Euclidean distance.

(10) $$Q_{ij}^{\prime }=Q_{ij}+\phi \left(Q_{ij}-Q_{kj}\right)$$

*φ* is a random number between [0, 1]. *Q* is one of the QoS parameters in the selected service *i* in task *j. K* is a randomly chosen number *k* ∈ {1, 2,..., *n*} (*I ≠ k*)*. N* is the total number of services in task *j*. For instance, the reliability component $R_{ij}^{\prime }$ is calculated by:

(11) $$R_{ij}^{\prime }=R_{ij}+\phi \left(R_{ij}-R_{kj}\right)$$

After calculating all four parameters in this way, we get a QoS vector ($A_{ij}^{\prime }$, $R_{ij}^{\prime }$, $C_{ij}^{\prime }$, $E_{ij}^{\prime }$). These parameters represent a virtual service, and the most similar service to the virtual service in the same task should be found. In order to find the most similar service, we use Euclidean distance, as in (12), between the virtual service $V_{ij}$ and candidate services $S_{ij}\;$in the same task. Our answer is the service with the smallest distance with the virtual service. This method is called the Approximate-Mapping local search method [77].

(12) $$D(S_{ij}\;,V_{ij})=\sqrt{\begin{array}{c} {\left(A_{ij}-A_{ij}^{\prime }\right)}^{2}+{\left(R_{ij}-R_{ij}^{\prime }\right)}^{2}+\\ \;{\left(C_{ij}-C_{ij}^{\prime }\right)}^{2}+{\left(E_{ij}-E_{ij}^{\prime }\right)}^{2} \end{array}}$$

Finally, the EBees compare the fitness of the original service against the obtained service and choose the better service for the next time’s local search.

Step 8: In this step, OBees are devoted to solutions for the local search based on probabilities given from solutions’ fitness. Solutions with better fitness have more chances of being selected. The probabilities are calculated by the following equation and are given to the roulette wheel to select solutions [75]:

(13) $$P_{i}=\frac{fit_{i}}{\sum_{n=1}^{SN}fit_{n}}$$

$fit_{i}\;$is equal to the fitness of *i*th solution *I* ∈ {1, 2, …, *S_N_*} chosen by EBees. *SN* is the total number of EBees in the population.

OBees carry out a local search on the selected solutions. The local search is carried out like the EBees local search in Step 7. If the fitness of the new solution is better than that of the old solution, the new one is replaced with the old one.

Step 9: If the fitness of a path cannot improve after a certain number, this path is deleted, and scout bees create a new path.

Step 10: The solution returns from the bee colony to the ant colony, and the pheromone is updated based on the fuzzy module; evaporation updates are carried out using the (14) [78]. After the EBees and OBees have performed their local searches, the solutions are returned to the ant colony. In this step, the pheromone levels on the paths are updated based on a fuzzy inference system. This system takes into account the fitness of the solutions and adjusts the pheromone levels accordingly. Additionally, evaporation updates are performed to decrease pheromone levels gradually.

(14) $$\tau_{ij\;}\left(t+1\right)\leftarrow \;\left(1-\rho \right)\times \tau_{ij}\left(t\right)$$

0< *ρ* ≤ 1 is the evaporation rate of the pheromone.

**Figure 5:**
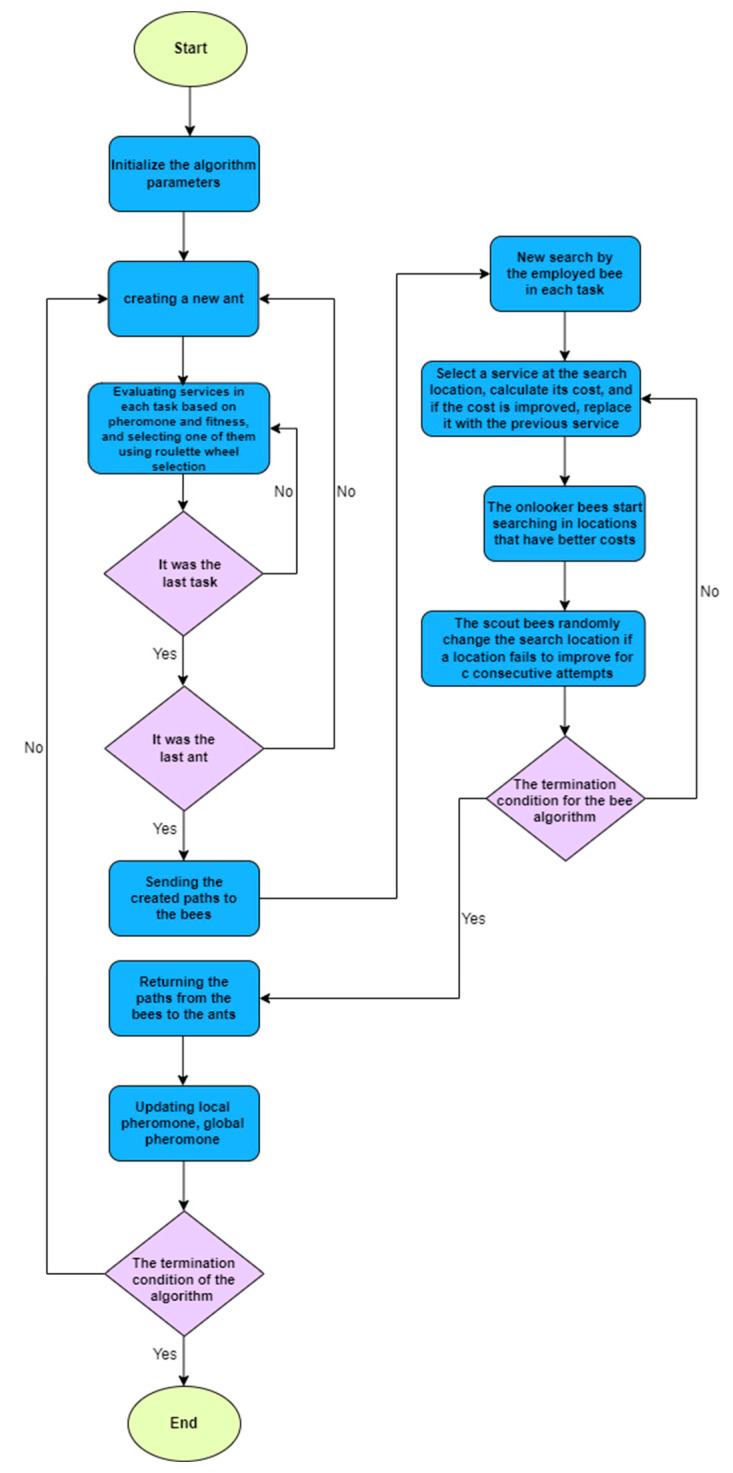
Flowchart of the proposed method.

Step 11: Go back to step 3 until the predetermined number of runs is attained. The pseudo-code (Algorithm 1) of the method and flowchart (Figure 5) is illustrated as follows:
**Algorithm 1: Proposed Method:**Initialized the algorithm’s parameters **While** (the termination condition is reached)  **For** ant = 1: population   Lunch an ant to construct a solution    **For** task = 1: the number of tasks     Calculation of the probability of each service      Selecting a service by a roulette wheel    **End for**  **End for**  The paths are given to EBees as initial solutions  **While** (the termination condition is reached)  EBees start to carry out the local search for solutions  Onlooker Bees select solutions based on their probability of local search  Scout Bees create a new solution instead of the solution that has not been improved  **End while**  EBees solution returns to ant colony optimization  Pheromones are updated based on the fuzzy system  Evaporation update is carried out **End while**

## 4. Experiment Results

This section describes the simulation tools, the data set, and the results obtained.

### 4.1. Simulation Tools and Dataset

The simulation is carried out utilizing a CPU core i7 2.4 GHz (4GByte RAM). The simulator program is Matlab R2017a. Matlab is used to simulate metaheuristic algorithms in many papers [46,79], and it is known as one of the best tools. Unfortunately, there are no multiple datasets available on the service composition in IoT domains, and the available datasets are confined to three datasets, QWS [80], WS-DREAM [81], and OWLS-Xplan [82], and datasets that are generated randomly. We considered the QWS dataset [83], similar to the work of some other scholars [41,84], to approve and validate our theory. The QWS dataset comprises a set of QWS measurements for 2507 service implementations. To tackle the issue of QoS value fluctuation during service runtime in dynamic IoT environments, QoS values are updated randomly after each service iteration by multiplying each QoS value with a random number [0.9, 1.1]. The energy model proposed in [85] can be studied to become acquainted with battery-operated objects’ energy consumption. This paper assumes that every device possesses a maximum battery charge (Cmax) and an initial charge (Cinitial). The amount of Cmax is 1500 mA.h, and Cinitial is randomly created in the range of 0.7 Cmax to 1.0 Cmax. Furthermore, if any device’s energy level drops below Cthroud (0.3 Cmax), the device will no longer provide any services and will not contribute to the composition process. In addition, a specific amount of power is consumed in every run, and this value is subtracted from the battery level of the device providing services.

### 4.2. Obtained Results

The three strategies presented in the articles [46,54,55]. are compared with our proposed method. Our method, a fuzzy-based hybrid algorithm with ACO-ABC, leverages fuzzy logic and optimization techniques to find an optimal service composition based on various QoS factors. Fuzzy logic enables handling uncertainty and imprecise information, while the ACO-ABC optimization approach aims to strike a balance between exploration and exploitation in the search space. This algorithm effectively considers multiple QoS factors simultaneously and finds a trade-off solution. In contrast, the FSCA-EQ approach [46] adopts a hierarchical optimization approach. It employs the compromise ratio method (CRM) to pre-select services that meet the user’s QoS requirements. The optimal service is then chosen based on relative dominance, considering energy profiles, QoS attributes, and user preferences. However, the FSCA-EQ approach for IoT service composition has some disadvantages, including:

1. Limited consideration of dynamic user demands: One of the drawbacks of the FSCA-EQ approach is its lack of explicit consideration for dynamic user demands. As user requirements change over time, the approach may not adapt effectively, potentially leading to suboptimal service composition.
2. Complexity of hierarchical optimization: The hierarchical optimization mechanism used in FSCA-EQ adds complexity to the service composition process. Managing and implementing this hierarchical approach can become challenging, especially when dealing with numerous IoT components and services.
3. Lack of flexibility in service selection: FSCA-EQ relies on the relative dominance concept for selecting the optimal service in the composite service. While it considers energy profiles, QoS attributes, and user preferences, this approach may limit the flexibility of service selection. It could overlook certain services that, although not dominant, could contribute to a more optimal composition.
4. Limited adaptation to changing IoT environments: The FSCA-EQ approach may face difficulties in adapting to dynamic changes in the IoT environment. As the IoT landscape evolves, new services may become available or existing ones may become obsolete. FSCA-EQ may not effectively handle such changes and might require manual adjustments or updates to its selection criteria.
5. Potential bias in service selection: The use of relative dominance and specific selection criteria in FSCA-EQ may introduce biases in the service composition process. Depending on how these criteria are defined and weighted, certain services or attributes may receive preferential treatment, potentially leading to imbalanced or suboptimal composite services.

Naseri and Navimipour [54] presented a new hybrid method for efficient service composition in cloud computing. The primary objective is to select suitable services based on QoS parameters while optimizing resource allocation. The proposed method combines an agent-based approach with the PSO algorithm to compose services and identify the best services based on a fitness function. However, some potential disadvantages of this approach for service composition in cloud computing could include:

1. Complexity and implementation challenges: Implementing a hybrid method that combines agent-based approaches with optimization algorithms like PSO can be complex [86]. Developing and deploying the algorithm effectively may require specialized expertise and resources.
2. Challenges in the distribution factor of importance: The method may face challenges when the distribution factor of importance is significant. In such cases, where the distribution of data centers is crucial, the method’s performance may be affected.
3. Lack of adaptability to dynamic environments: The proposed method may struggle to adapt to dynamic changes in the cloud environment, such as varying workloads, service availability, or QoS requirements. It may not possess real-time adaptation capabilities, limiting its responsiveness to dynamic service composition needs.
4. Sensitivity to fitness function and parameters: The effectiveness of the hybrid method heavily relies on the design and selection of the fitness function and parameter values. The algorithm’s performance may vary significantly depending on the chosen metrics and their weights, requiring careful tuning and experimentation.

One potential disadvantage of the method proposed by Chen, Wang [55] is its focus on a specific aspect of web service composition selection, primarily centered around QoS satisfaction degree as the primary objective. Some potential disadvantages of this approach for service composition include:

1. Limited scope: The method concentrates on optimizing QoS satisfaction degree as the primary objective, which might overlook other essential considerations, such as energy efficiency, cost-effectiveness, or security. Ignoring these factors could lead to suboptimal service compositions in scenarios where different aspects are equally critical.
2. Domain specificity: The effectiveness and feasibility of the proposed solution could vary depending on the specific context and the range of web services available. The genetic algorithm’s performance and adaptability might differ based on the characteristics and diversity of the available services, making it less suitable for certain application domains.
3. Algorithm parameter tuning: Genetic algorithms often require careful parameter tuning to achieve optimal results. The effectiveness of the proposed approach could be sensitive to the selection of genetic algorithm parameters, making it crucial to fine-tune these settings for each application scenario.
4. Computational complexity: Genetic algorithms can be computationally intensive, especially when dealing with a large number of web services and complex service composition scenarios. As the size of the search space increases, the time and resources required for optimization may become significant.
5. Limited multi-objective consideration: While the genetic algorithm is used for multi-objective optimization, the method primarily focuses on QoS satisfaction degree as the primary objective. It may not handle other competing objectives or trade-offs effectively, potentially limiting the range of service composition solutions [87,88].
6. Lack of real-world validation: Although the authors present test results indicating the feasibility and effectiveness of their solution, a comprehensive real-world validation might be necessary to assess the method’s performance and generalizability across diverse scenarios and user preferences.

The fuzzy-based hybrid algorithm with ABC and ACO offers several advantages over using PSO and GA for optimization tasks, especially in the context of service composition. Here are some key advantages:

1. Handling uncertainty: Fuzzy logic, which is incorporated into the fuzzy-based hybrid algorithm, allows for handling uncertainty and imprecise information. In service composition, where QoS parameters and user preferences may be ambiguous, fuzzy logic can provide better decision-making capabilities by considering linguistic variables and fuzzy rules. PSO and GA, on the other hand, do not inherently address uncertainty in the optimization process.
2. Comprehensive exploration and Exploitation: The hybrid nature of the fuzzy-based algorithm combines both ABC and ACO techniques. ACO is excellent at exploring the solution space to find optimal paths, while ABC excels at exploitation to refine the solutions found. This combination allows the algorithm to conduct a more comprehensive search, potentially leading to better-quality solutions compared to PSO and GA, which may focus more on exploration or exploitation alone.
3. Efficient convergence: The fuzzy-based hybrid algorithm’s ability to exploit the strengths of both ABC and ACO algorithms can result in more efficient convergence. By leveraging the synergistic effects of the two techniques, the algorithm may find optimal solutions more quickly, especially for complex optimization problems like service composition.
4. Consideration of multiple QoS factors: The fuzzy-based hybrid algorithm can effectively handle multiple QoS factors simultaneously, considering their interdependencies. This careful consideration of various QoS parameters ensures a more balanced and optimal composition of services. PSO and GA may face challenges in efficiently managing multiple objectives in the optimization process.
5. Better adaptability: The fuzzy-based hybrid algorithm with ABC and ACO may exhibit better adaptability to dynamic changes in the optimization landscape. As service requirements or constraints change, the algorithm can adjust its search strategy more effectively compared to PSO and GA, which may require more manual parameter tuning.
6. Reduced sensitivity to parameters: The fuzzy-based approach typically involves fewer parameters requiring tuning than PSO and GA. This reduces the algorithm’s sensitivity to parameter settings and simplifies optimization.
7. Increased robustness: The hybridization of ABC and ACO techniques adds robustness to the fuzzy-based algorithm. Combining two complementary approaches makes the algorithm less likely to get trapped in local optima, leading to more robust and globally optimal solutions.

**Figure 6:**
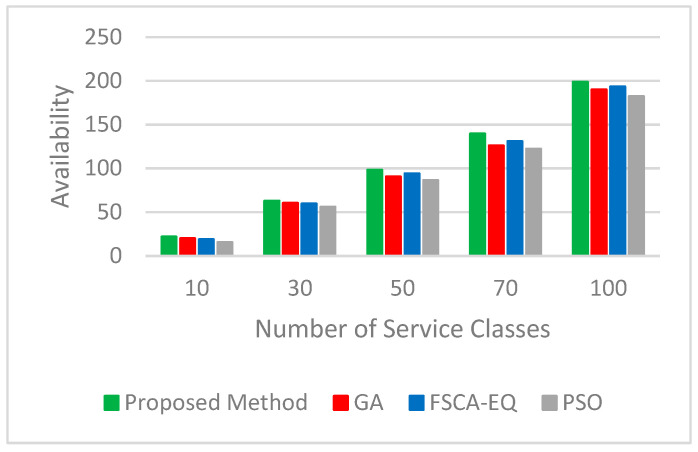
Availability for 10, 30, 50, 70, and 100 service classes with 50 candidate services.

**Figure 7:**
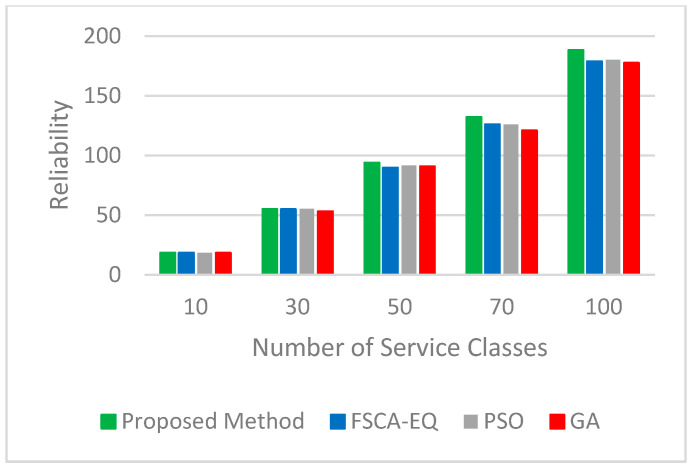
Reliability for 10, 30, 50, 70, and 100 service classes with 50 candidate services.

**Figure 8:**
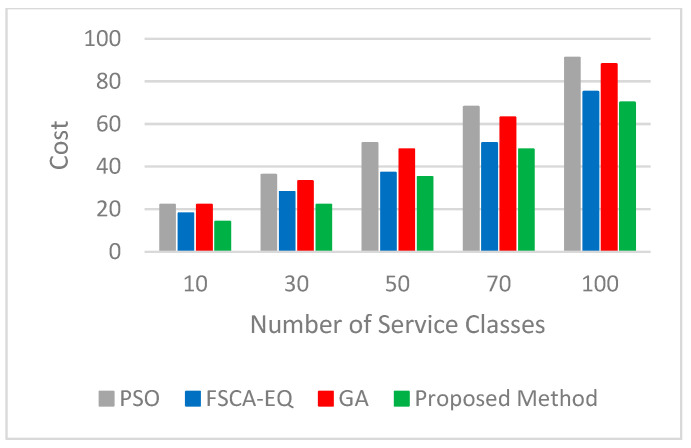
Cost for 10, 30, 50, 70, and 100 service classes with 50 candidate services.

**Figure 9:**
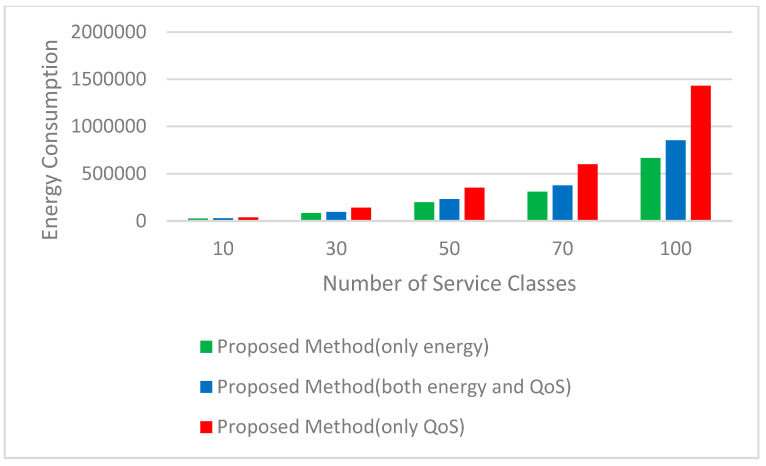
Energy consumption for 10, 30, 50, 70, and 100 service classes with 50 candidate services.

**Figure 10:**
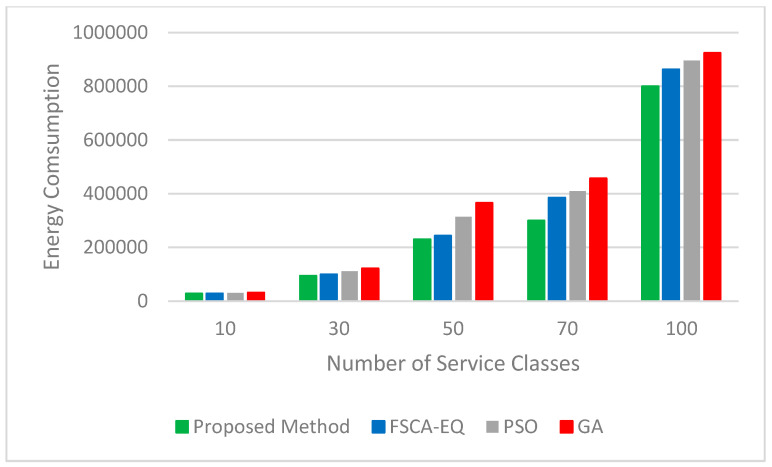
Energy consumption for 10, 30, 50, 70, and 100 service classes with 50 candidate services.

Four important parameters of the quality of services, such as availability, reliability, energy, and cost, are considered. The results validate that the proposed method has great performance. We utilized 10, 30, 50, 70, and 100 service classes (each class is related to a task) with 50 candidate services to assess the quality of service in simulation tests. Figure 6 and Figure 7, respectively, represent the logarithm (base 10) of the results obtained for the real value of availability and reliability parameters. As can be seen, in all three parameters mentioned, the proposed method has achieved good results. In Figure 8, the figure for the cost parameter of the proposed approach is smaller than that of other algorithms. When the number of requests goes up, this parameter declines dramatically. The reason for this issue is rooted in the optimal selection of services in the proposed algorithm. In our research, two scenarios to evaluate the energy parameter are considered. In the first one (Figure 9), the proposed method, considering both the energy profile and QoS (cost, availability, and reliability) in the selection process, is compared with the same method considering either the energy profile or QoS. In Figure 10, which illustrates the second scenario, the energy parameter in the proposed method is compared with the mentioned algorithms [46,54,55]. As shown in Figure 9, the amount of the energy consumed by the method when both energy and QoS are considered is close to that of when energy is only considered, and it is just between 12% and 28% higher than that. In addition, the energy consumption of the proposed method when both energy and QoS are considered is from 42% to 68% lower than that when QoS is only considered.. Table 3 shows the improvement percentages of energy, cost, reliability, and availability in the proposed method (both QoS and energy are considered) compared with the same parameters in FSCA-EQ, GA, and PSO.

## 5. Conclusions and Future Work

This section discusses the accomplishments and summary of the paper, along with future investigation tips for IoT in various fields. The importance of IoT in pervasive computing is undeniable, as embedded devices are increasingly prevalent in all aspects of life, even interpersonal interactions. These devices generate data that need to be processed and combined to extract meaningful insights. This paper presents a novel fuzzy-based hybrid algorithm that combines an ACO algorithm and an ABC algorithm for IoT service composition. Initially, the service composition problem is transformed into finding an optimal path with specific QoS requirements in a directed acyclic graph. The hybrid algorithm effectively leverages the strengths of both the ACO and ABC algorithms to obtain the optimal solution. The analysis and experiments demonstrate that this hybrid approach outperforms the individual ACO and ABC algorithms in terms of efficiency and flexibility. Our algorithm efficiently combines IoT services based on their QoS, surpassing the ant colony and bee colony optimization algorithms, and effectively fulfills user requests. Additionally, the proposed method exhibits better reliability, availability, and cost-effectiveness. Given IoT deployments’ increasing complexity and scale, our research opens up new possibilities for QoS-aware service composition, leading to enhanced user experiences, improved system reliability, and sustainable IoT ecosystems.

However, some unresolved issues and intriguing areas still require further investigation. Presently, no mechanism addresses all aspects related to IoT service composition. While some methods consider QoS parameters such as reliability, response time, and convergence time, others ignore these critical factors. It is essential to evaluate additional quality criteria to enhance service composition performance. Furthermore, inter-service dependencies and conflicts are significant concerns in QoS-aware service composition, but only a few papers focus on addressing these issues. In some IoT-based service composition scenarios, service selection for each task occurs independently of other tasks, leading to potential conflicts and inefficiencies. Future research should explore incorporating different QoS parameters and addressing inter-service dependencies and conflicts to further enhance service composition methods in IoT applications. By addressing these challenges, we can create more robust and efficient IoT systems to meet the ever-growing demands of users and applications.

However, it is essential to acknowledge that technological constraints can challenge the service composition process. The selection of services in a composition heavily depends on various restrictions, such as time and location. Unfortunately, many IoT researchers have not thoroughly explored inter-service dependencies and conflicts. As such, the current study has some limitations that need to be addressed.

1. Scalability: The proposed algorithm’s scalability is not explicitly addressed. As the number of devices and services in the IoT ecosystem grows, the algorithm’s ability to handle larger-scale compositions may become a limitation. It is crucial to consider the method’s performance and efficiency when dealing with a large number of IoT devices and services.
2. Real-world Deployment: The practical aspects of implementing the proposed approach in real-world IoT systems are not discussed. It would be valuable to address the compatibility, interoperability, and deployment challenges that may arise when integrating cloud and fog computing infrastructures.

For future research, the following directions can be considered:

1. Extension of QoS Parameters: Future work could focus on expanding the QoS parameters considered in the service composition process. Investigating additional metrics related to service quality, such as security [89,90], privacy [91,92], and network bandwidth, would provide a more comprehensive evaluation and improve the performance of the service composition method.
2. Inter-Service Dependencies and Conflicts: Addressing inter-service dependencies and conflicts in IoT service composition should be a priority. Developing techniques or algorithms that explicitly handle conflicts between service compositions and address the challenges posed by inter-service dependencies would be beneficial.
3. Real-World Implementation and Performance Evaluation: Testing the proposed method via implementation in a real IoT application would provide valuable insights into its practicality and performance in a realistic setting.
4. Edge Computing Optimization: Considering the emerging paradigm of edge computing, future work could explore optimizations that leverage the potential of processing IoT applications at the edge networks near the devices. Investigating how the proposed service composition method can be enhanced or adapted for edge computing environments would be valuable.
5. Combination of the applied algorithm with some powerful techniques: In many cases, the hybrid algorithms have delivered good results. Therefore, we can combine the proposed algorithm with some other algorithms, such as the greedy algorithm [93,94,95], active subspace random optimization [96], neural networks [97], and deep/federated/machine learning [98,99,100].

Finally, the main novelty and impact of the current research are:

1. Comparison with state-of-the-art methods: To assess the impact and novelty of the research, a detailed comparison of the proposed hybrid algorithm for service composition with recently introduced methods is necessary. This analysis would help determine the advancements and improvements achieved by the proposed approach.
2. Integration of fog computing: The proposed cloud-/fog-based service composition approach acknowledges the emergence of fog computing as a paradigm to process IoT applications at the edge networks. Assessing the benefits and performance enhancements achieved via this integration would further highlight the novelty of the research.

## Figures and Tables

**Table 1:** Summary of related works.

Reference	Methodology	Strengths	Limitations
Asghari, Rahmani [41]	Hybrid algorithm (GA + SFLA)	Privacy-aware service composition Computational model for privacy level Scalability Improved user satisfaction	Lack of consideration for energy, security, and lightweight encryption Complexity in implementation Computational overhead Parameter tuning Limited real-world data validation
Sefati and Navimipour [50]	ACO algorithm + Hidden Markov model	Flexible service composition Efficient cost reduction Robustness and reliability	Inefficiency in energy optimization Computational complexity Parameter tuning Limited validation Scalability issues Overfitting
Souri and Ghobaei-Arani [51]	Formal verification + Whale Optimization Algorithm	Flexibility Scalability Resource efficiency	Slow convergence Algorithm complexity Data privacy and security concerns Overhead and latency Lack of energy consumption consideration
Chai, Du [46]	Hierarchical optimization	Composite service flexibility Resource efficiency Real-time decision making	High computational cost Limited details on implementation Scalability challenges Algorithm complexity
Ibrahim, Rashid [52]	Shuffled Frog Leaping Algorithm (SFGA)	Faster service selection Effective composition in service cost and response time Improved connectivity Enhanced productivity Convenience	Lack of consideration for reliability, availability, and other important IoT parameters Privacy and security risks Dependency on infrastructure Information overload
Jian, Li [53]	Modified Bird Swarm Optimization Algorithm (MBSA)	Improved global search ability Efficient execution time Multi-objective optimization Enriched diversity	Computational complexity Scalability Lack of guaranteed optimality
Guzel and Ozdemir [45]	NSGA-II-based model	Energy consumption optimization Fairness among IoT services Time window optimization Improved energy efficiency without QoS degradation	Computational complexity Scalability challenges
Naseri and Navimipour [54]	Agent-based + PSO	Resource optimization Scalability Reduced waiting time Improved performance	Complexity of the hybrid method Limited distribution factor handling
Chen, Wang [55]	Genetic Algorithm	Enhanced service quality Multi-objective optimization Time efficiency	Scalability challenges Local optima traps
Ullah, Ali [56]	Multi-Objective Genetic Algorithm + Demand response programs	Carbon emission reduction Renewable energy integration Multi-objective optimization Uncertainty management	Implementation costs Complexity in DRP participation Scalability issues Data privacy and security
Ullah, Khan [57]	Multi-Objective Genetic Algorithm + Decision-Making Mechanism	High flexibility Multi-objective optimization Non-dominated solutions	Computational complexity Data privacy and security concerns Communication and control overhead
Ali, Ullah [58]	Multi-Objective Wind-Driven Optimization	Energy efficiency Reduced operational costs Adaptability and scalability	Complexity Sensitivity to input data Limited flexibility High implementation costs
Hafeez, Wadud [59]	Wind-Driven Bacterial Foraging Algorithm	Energy efficiency Cost reduction User comfort	Reliability concerns Complexity Limited compatibility

**Table 2:** QoS aggregation functions for composite services.

QoS Attributes	Aggregation Function
Availability	q_a_ (S) = $\overset{n}{\underset{i=1}{\prod }}q$_a_ (s_i_)
Reliability	q_r_(S) = $\overset{n}{\underset{i=1}{\prod }}q$_r_ (s_i_)
Cost	q_c_ (S) = $\overset{n}{\underset{i=1}{\sum }}q$_c_ (s_i_)
Energy	q_e_ (S) = $\overset{n}{\underset{i=1}{\sum }}q$_e_ (s_i_)

**Table 3:** The improvement percentage.

	Availability	Reliability	Cost	Energy
The Proposed Method Compared to FSCA-EQ	5.02%	4.22%	9.68%	10.33%
The Proposed Method Compared to GA	7.14%	5.89%	25.60%	23.55%
The Proposed Method Compared to PSO	12.68%	3.45%	29.48%	17.46%

## Data Availability

All data are reported in the paper.
